# Lupus Nephritis in a Patient with Sickle Cell Disease

**DOI:** 10.1155/2013/907950

**Published:** 2013-11-07

**Authors:** Vinay Minocha, Fauzia Rana

**Affiliations:** ^1^Department of Internal Medicine, University of Florida College of Medicine, Jacksonville, USA; ^2^Department of Oncology, University of Florida College of Medicine, Jacksonville, USA

## Abstract

*Introduction*. The diagnosis of systemic lupus erythematosus (SLE) in patients with sickle cell disease (SCD) can be difficult to establish because the musculoskeletal, central nervous system, and renal manifestations are similar in both diseases. In the presented case, we highlight the diagnostic challenge that can evolve in patients with a concurrence of both diseases and we establish the importance of early recognition and treatment of lupus nephritis in patients with SCD. *Case Presentation*. We present a case of a 31-year-old African American female with sickle-C disease (hemoglobin SC) who was admitted to our hospital with complaints of periumbilical abdominal pain associated with intractable nausea and vomiting, abdominal distension, and worsening lower extremity edema. Urine studies revealed nephrotic range proteinuria and the immunological investigations were consistent with lupus. A renal biopsy revealed focal proliferative lupus nephritis. *Conclusion*. It is important to consider the presence of a coexisting autoimmune disease in a patient with sickle hemoglobinopathy who displays an atypical and multisystem presentation that is unresponsive to conventional therapies. When a significant kidney disease is present, a renal biopsy is critical in identifying the etiology of a renal abnormality in the setting of coexisting SLE and SCD.

## 1. Background

Systemic lupus erythematosus (SLE), or lupus, is a chronic, progressive, autoimmune disorder that affects multiple organ systems, with a broad range of clinical and laboratory manifestations [1]. Sickle cell disease (SCD) encompasses a group of autosomal-recessive genetic disorders characterized by the production of abnormal hemoglobin S (HbS). The protean clinical features of SCD result from chronic variable intravascular hemolysis and microvascular ischemia, leading to damage in multiple organs [2]. The diagnosis of SLE in patients with an underlying chronic hemoglobinopathy can be difficult to establish because the musculoskeletal, central nervous system, and renal manifestations are similar in both diseases. In the presented case, we highlight the diagnostic challenge that can evolve in patients with a concurrence of both diseases and we establish the importance of early recognition and treatment of lupus nephritis in patients with SCD.

## 2. Case Presentation

A 31-year-old African American female with sickle-C disease (hemoglobin SC) was admitted to our hospital with complaints of periumbilical abdominal pain associated with intractable nausea and vomiting, abdominal distension, and worsening lower extremity edema. She was admitted a month previously for similar symptoms but was discharged after her symptoms abated and were attributed to a viral gastroenteritis. Her history was also significant for intermittent episodes of joint swelling predominantly in the small joints of the hands, knees, and ankles. On physical examination, the patient was pale and dehydrated. She had a temperature of 98.4 F, her heart rate was 109 beats/minute, and her blood pressure was 155/90 mmHg. Her abdomen was mildly distended with diffuse abdominal tenderness on palpation. There was no organomegaly/scars and normal bowel sounds were present. Cardiorespiratory and neurological examination were unremarkable. 

Blood test results showed a normocytic anemia at 8.8 gm/dL with a normal total leukocyte count of 9,100/cmm and a normal platelet count at 241 K/microL. Her liver function tests, renal function tests, and lipase and amylase levels were unremarkable. Hemoglobin (Hb) electrophoresis test results showed Hb S at 53.1 percent, Hb C at 46.9 percent, and Hb A1 at 0 percent, confirming a diagnosis of SCD (hemoglobin S/C). A computed tomography scan (CT) of the abdomen and pelvis showed nonspecific small bowel wall and colonic edema. In addition, her CT scan revealed bilateral pleural effusions, a pericardial effusion, ascites, and body wall edema. Ultrasonography showed ascites and increased echogenicity of the kidneys with maintained corticomedullary differentiation. A subsequent esophagogastroduodenoscopy (EGD) and colonoscopy were normal. Based on her radiographic findings, the major considerations at this point included SCD-related vasoocclusion involving the bowel, functional asplenia with subsequent infection secondary to SCD, an infectious enteritis, or inflammatory bowel disease. She was therefore started on parenteral antibiotic and fluid therapy. 

During the course of her admission, she was found to have progressive pedal and periorbital edema, worsening distension of the abdomen, and increased dyspnea on exertion. An echocardiogram subsequently confirmed the presence of the previously visualized small-to-moderate pericardial effusion. The patient had no evidence of tamponade and had normal systolic left ventricular function with a normal ejection fraction. A urinalysis revealed proteinuria and a formal 24 hour urine study yielded 4.5 g protein with a spot urine protein : creatinine ratio of 3.8 consistent with nephrotic range proteinuria. 

Immunological investigations revealed a positive anti-nuclear antibody (ANA) with a 1 : 640 titer in a speckled pattern. Anti-DNA antibody (dsDNA) was positive at 46 (normal: 0–9) with positive anti-Smith antibodies and low C3 (14 mg/dL) and C4 (2.7 mg/dL). Anticardiolipin and Beta 2 glycoprotein antibodies were negative. Tests for cryoglobulins and anti-neutrophil cytoplasmic antibodies (ANCA) were also negative. Serum immunofixation was normal and urine immunofixation revealed albuminuria with no evidence of a monoclonal paraprotein. Serology test results for human immunodeficiency virus, hepatitis B, and hepatitis C were negative.

**Figure 1:**
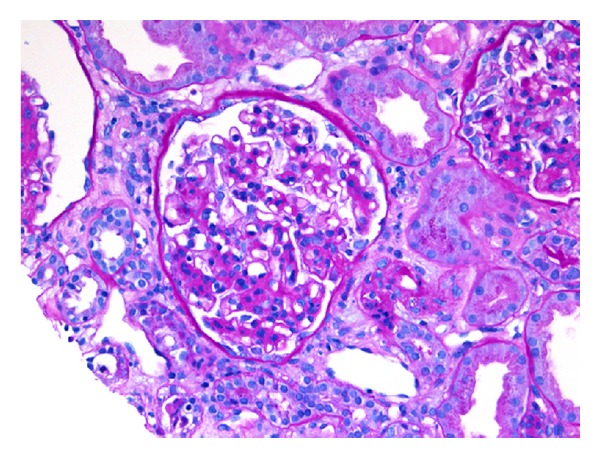
One glomerulus showing mesangial hypercellularity and mesangial matrix expansion; PAS stain, ×20.

**Figure 2:**
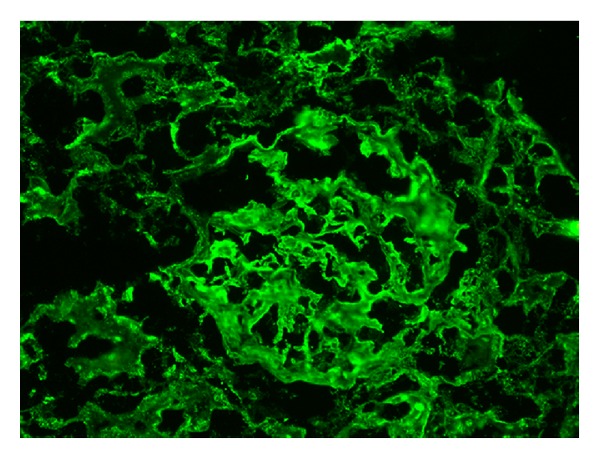
Immunofluorescence. 3+ positivity. Mesangial IgG immune complex deposition.

**Figure 3:**
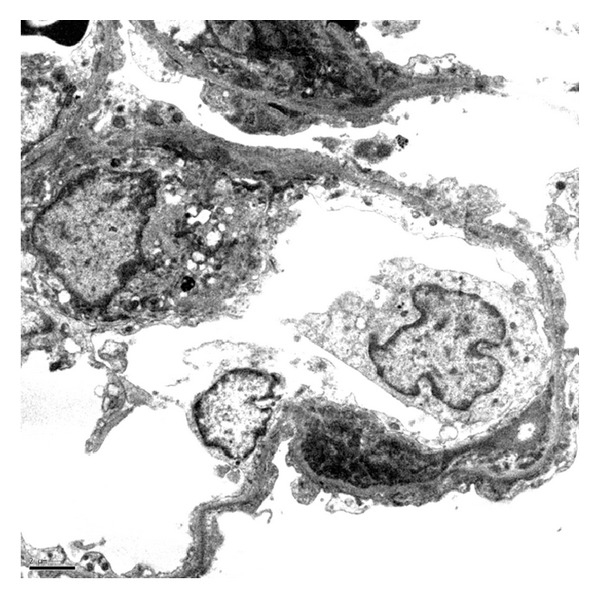
Electron Microscopy. Mesangial and subendothelial immune complex deposition.

A diagnosis of SLE in a patient with SCD was established, with five of the diagnostic criteria of the American College of Rheumatology being met. A renal biopsy revealed focal proliferative lupus nephritis, World Health Organization (WHO) class III, and International Society of Nephrology/Renal Pathology Society (ISN/RPS) class III(A), with mild activity and essentially no chronicity. The specimen was remarkable for mesangial hypercellularity and mesangial immune complex deposition (See Figures 1, 2, and 3).

Steroids were administered as a pulse of methylprednisolone in conjunction with cyclophosphamide. Her symptoms quickly improved. She was eventually discharged on prednisone and azathioprine. At her 18-month follow up, her lupus nephritis was in clinical remission on prednisone 15 mg per day, hydroxychloroquine, and mycophenolate mofetil; she had not experienced a sickle cell crisis this time.

## 3. Discussion

Our case describes an African-American woman with a diagnosed history of Sickle Cell Disease (SCD) who goes on to develop Systemic Lupus Erythematosus (SLE) and in particular Lupus Nephritis. The current literature indicates that the association between SLE and SCD is uncommon [3]. Most of the information concerning the clinical presentation and management of patients with an overlap of these two distinct clinical entities has been derived from case reports. As yet, no large prospective epidemiological studies have been conducted to determine whether there is an increased prevalence of autoimmune disease in patients with SCD [4].

As described in this case, the recognition of SLE is often difficult and delayed when there is a coexistence of both diseases. The diagnosis of SCD is usually made several years prior to the onset of SLE. Both are chronic, progressive diseases with diverse multisystem manifestations that show a significant overlap in presentation. This accounts for the diagnostic difficulty that may arise with the concurrence of SLE and SCD [5]. Furthermore, approximately 20% of SCD patients have positive ANA antibodies with titers greater than 1/160, making the diagnosis more challenging in clinical practice [6]. 

In our patient, her gastrointestinal symptoms were initially attributed to SCD-related vasoocclusion involving the bowel and her musculoskeletal complaints were associated with a similar etiology. A review of published cases indicates that in patients with SCD, articular involvement is the most frequent lupus-related symptom, present in 84 percent of the cases, followed by serositis (36 percent), and glomerulonephritis class III or IV (11 percent) [7]. 

Our patient complained of intermittent episodes of joint swelling and pain predominantly in the small joints of the hands, knees, and ankles. Patients with SCD typically present with a noninflammatory monoarticular arthritis of short duration (14 days). In contrast, the arthritis of SLE is characteristically symmetric, polyarticular, and nonerosive. The patient's presentation was therefore more consistent with SLE. In cases where the clinical presentation is less distinct, synovial biopsies may provide added confirmatory evidence. In SCD, synovial biopsy demonstrates focal proliferation, few chronic inflammatory cells, and microvascular thrombosis resulting from vascular occlusion by sickled erythrocytes. In SLE, synovial biopsies show diffuse proliferation and minimal destruction of cartilage or bone [8]. As in this case, patients who present with the less severe articular manifestations of SLE may be diagnosed with vasoocclusive crises.

Radiographic studies and echocardiography in this patient confirmed the presence of a serositis as demonstrated by a pericardial effusion, bilateral pleural effusions, and ascites. Chest pain, pulmonary infiltrates, cardiomegaly, and congestive heart failure may be seen with both SCD and SLE [9]. The abdominal pain associated with SLE may be diagnosed as the vasoocclusive abdominal crisis seen in SCD. Multisystem findings not explained by SCD alone should prompt a search for an underlying connective tissue disease such as SLE. According to a case report published by Shetty and colleagues, pericarditis in patients with SCD should be a clue for workup of collagen vascular disease as well as infectious process [10].

The frequency and titers of antibodies in SCD have been reported as relatively higher than in population controls regardless of the presence of autoimmune clinical signs [6]. It has been hypothesized that in SCD, autoantibodies could be induced by a chronic inflammatory state stemming from chronic intravascular and extravascular hemolysis. In an environment of rapid cell turnover, autoantibodies against self-components could be produced. Moreover, environmental stimuli, such as recurrent infections in a permanent inflammatory background, are likely to trigger the production of autoantibodies. Another hypothesis is that the dysfunctional immune status of patients with this disease arising from functional hyposplenia, complement pathway defects, and impairment of opsonization and phagocytosis could impede clearance of immune complexes [6]. Therefore it has been proposed that when SLE is suspected in a patient with SCD, the best serologic markers appear to be SLE-specific autoantibodies such as anti-dsDNA and anti-Smith. Persistent hypocomplementemia also supports the presence of an immune complex-mediated disease [8]. In the case described, the patient had marked hypocomplementemia in association with positive SLE-specific antibodies further substantiating her diagnosis of SLE.

Our patient eventually showed clinical manifestations of nephrotic syndrome including pedal and periorbital edema, distension of the abdomen, and exertional dyspnea. Urine studies showing nephrotic range proteinuria (>4.5 g protein/24 hrs) in association with positive dsDNA antibodies and hypocomplementemia suggested that the patient had ongoing lupus nephritis. This diagnosis was confirmed by a renal biopsy subsequently showing Focal Proliferative Lupus Nephritis, Class III. 

Progressive renal injury is prominent in both SCD and SLE. SCD can present with nephrotic syndrome, and four different types of glomerulopathy have been described in SCD: Focal Segmental Glomerulosclerosis (FSGS), membranoproliferative glomerulonephritis (MPGN), glomerulopathy specific to SCD, and thrombotic microangiopathy (TMA). Regardless of which type of glomerular damage is present, all renal biopsies from SCD patients show hypertrophied glomeruli with distended capillaries due to the sickled blood cells, which is described as a glomerulopathy specific to SCD [11]. Microalbuminuria is a sensitive marker of sickle nephropathy that precedes proteinuria and increases in prevalence with age [9]. This may progress to nephrotic syndrome and ultimately to renal failure, a common cause of mortality among adults with SCD [11].

Differentiating sickle nephropathy and lupus nephritis is difficult without a renal biopsy. Interestingly, varying degrees of immune complex deposition can be found in sickle cell nephropathy especially in MPGN [8]. Furthermore, mesangial expansion and basement membrane duplication seen in SCD can delay the pathologic diagnosis unless it is made in conjunction with serologic workup [12]. Identifying the etiology of a renal abnormality in the setting of coexisting SLE and SCD is important, because there are different implications for morbidity, mortality, and therapeutic options. In most cases (68%), sickle cell glomerulopathy continues to progress into chronic renal failure [11]. In principle, this progression can only be slowed or halted by any therapies which limit the frequency and severity of red blood cell sickling. In contrast, renal involvement in SLE often responds to immunosuppressive therapy.

## 4. Conclusion

The above report demonstrates the importance of considering the presence of a coexisting autoimmune disease in a patient with sickle hemoglobinopathy who displays an atypical and multisystem presentation that is unresponsive to conventional therapies. The presence of SLE-specific antibodies and hypocomplementemia can be especially helpful in substantiating a diagnosis of lupus. Finally, when significant kidney disease is present, a renal biopsy is critical in establishing the correct diagnosis. Identifying the etiology of a renal abnormality in the setting of coexisting SLE and SCD is important, because there are different implications for morbidity, mortality, and therapeutic options.

## References

[1] Gurevitz SL, Snyder JA, Wessel EK, Frey J, Williamson BA (2013). Systemic lupus erythematosus: a review of the disease and treatment options. *The Consultant Pharmacist*.

[2] Michel M, Habibi A, Godeau B (2008). Characteristics and outcome of connective tissue diseases in patients with sickle-cell disease: report of 30 cases. *Seminars in Arthritis and Rheumatism*.

[3] Cherner M, Isenberg D (2010). The overlap of systemic lupus erythematosus and sickle cell disease: report of two cases and a review of the literature. *Lupus*.

[4] Katsanis E, Hsu E, Luke K-H, McKee JA (1987). Systemic lupus erythematosus and sickle hemoglobinopathies: a report of two cases and review of the literature. *American Journal of Hematology*.

[5] Saxena VR, Mina R, Moallem HJ, Rao SP, Miller ST (2003). Systemic lupus erythematosus in children with sickle cell disease. *Journal of Pediatric Hematology/Oncology*.

[6] Toly-Ndour C, Rouquette A-M, Obadia S (2011). High titers of autoantibodies in patients with sickle-cell disease. *Journal of Rheumatology*.

[7] Maamar M, Tazi-Mezalek Z, Harmouche H, Mounfaloti W, Adnaoui M, Aouni M (2012). Systemic lupus erythematosus associated with sickle-cell disease: a case report and literature review. *Journal of Medical Case Reports*.

[8] Khalidi NA, Ajmani H, Varga J (2005). Coexisting systemic lupus erythematosus and sickle cell disease: a diagnostic and therapeutic challenge. *Journal of Clinical Rheumatology*.

[9] Appenzeller S, Fattori A, Saad ST, Costallat LTL (2008). Systemic lupus erythematosus in patients with sickle cell disease. *Clinical Rheumatology*.

[10] Shetty AK, Baliga MR, Gedalia A, Warrier RP (1998). Systemic lupus erythematosus and sickle cell disease. *Indian Journal of Pediatrics*.

[11] López Revuelta K, Ricard Andrés MP (2011). Kidney abnormalities in sickle cell disease. *Nefrologia*.

[12] Kanodia KV, Vanikar AV, Goplani KR, Gupta SB, Trivedi HL (2008). Sickle cell nephropathy with diffuse proliferative lupus nephritis: a case report. *Diagnostic Pathology*.

